# Prevalence and Predictors of Malaria in Human Immunodeficiency Virus Infected Patients in Beira, Mozambique

**DOI:** 10.3390/ijerph15092032

**Published:** 2018-09-17

**Authors:** Francesco Di Gennaro, Claudia Marotta, Damiano Pizzol, Kajal Chhaganlal, Laura Monno, Giovanni Putoto, Annalisa Saracino, Alessandra Casuccio, Walter Mazzucco

**Affiliations:** 1 Department of Infectious Diseases, University of Bari “Aldo Moro”, 35128 Bari, Italy; cicciodigennaro@yahoo.it (F.D.G.); laura.monno@uniba.it (L.M.); annalisa.saracino@uniba.it (A.S.); 2 Department of Science for Health Promotion and Mother to Child Care “G. D’Alessandro”, via del Vespro, University of Palermo, 90217 Palermo, Italy; alessandra.casuccio@unipa.it (A.C.); walter.mazzucco@unipa.it (W.M.); 3 Doctors with Africa—CUAMM, Research Unit, Beira 1363, Mozambique; d.pizzol@cuamm.org; 4 Center for Research in Infectious Diseases, Faculty of Health Sciences, Catholic University of Mozambique, Beira 1363, Mozambique; kajal83bc@yahoo.com; 5 Research Section, Doctors with Africa CUAMM, 35128 Padova, Italy; g.putoto@cuamm.org

**Keywords:** malaria prevalence, HIV, HIV-malaria co-infection, malaria predictors in developing countries, cotrimoxazole prophylaxis, Mozambique

## Abstract

Co-infection between malaria and HIV has major public health implications. The aims of this study were to assess the malaria prevalence and to identify predictors of positivity to malaria Test in HIV positive patients admitted to the health center São Lucas of Beira, Mozambique. A retrospective cross-sectional study was performed from January 2016 to December 2016. Overall, 701 adult HIV patients were enrolled, positivity to malaria test was found in 232 (33.0%). These patients were found to be more frequently unemployed (76.3%), aged under 40 (72.0%), with a HIV positive partner (22.4%) and with a CD4 cell count <200 (59.9%). The following variables were predictors of malaria: age under 40 (O.R. = 1.56; 95%CI: 1.22–2.08), being unemployed (O.R. = 1.74; 95%CI: 1.24–2.21), irregularity of cotrimoxazole prophylaxis’s (O.R. = 1.42; 95%CI: 1.10–1.78), CD4 cell count <200 (O.R. = 2.01; 95%CI: 1.42–2.32) and tuberculosis comorbidity (O.R. = 1.58; 95%CI: 1.17–2.79). In conclusion, high malaria prevalence was found in HIV patients accessing the out-patients centre of São Lucas of Beira. Our findings allowed us to identify the profile of HIV patients needing more medical attention: young adults, unemployed, with a low CD4 cell count and irregularly accessing to ART and cotrimoxazole prophylaxis.

## 1. Introduction

Despite improvements, malaria and acquired immune deficiency syndrome (AIDS) are two of the most important infectious diseases worldwide [1]. Malaria is on the list of AIDS-related opportunistic infections and the highest occurrence of human immunodeficiency virus (HIV) and malaria cases are reported in sub-Saharan countries [2]. Particularly, HIV infection is expected to increase the morbidity and mortality attributed to malaria, reducing the immune response against *Plasmodium* spp. and, consequently, leading to a more frequent occurrence of clinically severe malaria cases [3]. Interestingly, the use of cotrimoxazole (CTX) prophylaxis and antiretroviral therapy (ART) in HIV-infected patients seems to provide a protective effect from malaria [4].

Mozambique is one of the sub-Saharan African countries with the highest incidences of HIV co-infection associated to endemic malaria [5,6]. In Mozambique, *Plasmodium falciparum* infection accounts for 90% of all malaria cases, followed by *P. malariae* and *P. ovale* responsible of about 9% and 1%, respectively. In 2015, the confirmed cases of malaria were 8,520,376 [5]. Furthermore Mozambique has one of the highest incidences of HIV worldwide with an estimated national prevalence of 12.5% in the age-group 15–49 and the estimated number of deaths of 62,000/years [6].

However, the number of studies on prevalence and clinical manifestations of HIV- malaria co-infection in Mozambique is limited [7,8]. The aims of our study were: (i) to verify the prevalence of malaria in HIV patients and (ii) to identify predictors of positivity to malaria test in HIV patients admitted to the health center of São Lucas of Beira, the second largest city of Mozambique.

## 2. Materials and Methods

A retrospective observational cross-sectional study was designed and implemented to analyze data of patients accessed the health centre of São Lucas of Beira, Sofala, Mozambique, from January 2016 to December 2016.

The health center of São Lucas provides access to free care and treatment of HIV/AIDS patients in an out-patients setting. For each new admitted patient, medical history is collected and the HIV status is checked with the HIV Rapid Test and, if positive, confirmed by western blot. Each consecutive consultation includes full clinical examination, (including HIV status according to WHO), ART therapy, cotrimoxazole prophylaxis, CD4+ T cell count, partner HIV status, co-morbidities (diabetes, hypertension and tuberculosis) and other sexual transmitted infections (STI), including genital herpes, condyloma, syphilis, gonorrhea and candidiasis. With regard to ART therapy and cotrimoxazole prophylaxis, information on regularity of their administration are also collected. In case of clinical suspicion of diabetes or tuberculosis, specific diagnostic protocol according to WHO guidelines is applied to confirm the diagnosis [9].

Malaria screening is performed using the Malaria Rapid Diagnostic Test (RDT) kit, as described by the manufacturer’s (Standard Diagnostics Bioline, 2013). The confirmation of RDT results is obtained by blood smear microscopy [10]. All patients with a positive malaria blood slide and/or rapid diagnostic test are considered as infected with malaria.

A sample size estimation was performed using the following formula [11]:

(1) $$n=\frac{Z^{2}x\;p\left(1-p\right)}{e^{2}}$$

where Z = 1.96, p = prevalence of malaria among people living with HIV (PLWHIV): 25.9% [12], e = error rate: 0.05.

A convenience sampling of at least 298 PLWHIV was estimated. All HIV/AIDS adult patients (>18 years) that consecutively accessed the health center for a medical consultation or to collect their antiretroviral (ARV) drugs from January 2016 to December 2016 were identified through the patients’ registry and were recruited in the study.

### 2.1. Statistical Analysis

Categorical variables were reported as absolute and relative frequencies (percentages). Chi-square test (with the Yates’ correction as required) was used to compare categorical variables. A logistic regression model was implemented as follows: Malaria positivity was considered as dependent variable and each one of the available factors at the baseline evaluation were used as independent variables (univariate analysis). In the multivariate analysis all the factors with a *p*-value < 0.10 at the univariate analyses were included. Multicollinearity among covariates was assessed through the variance inflaction factor (VIF), taking a value of 2 for excluding a covariate. However, no variable was excluded according to the previous criterion. Odds Ratios (ORs) as adjusted Odds Ratios (Adj-ORs) with their 95% confidence intervals (CIs) were used to measure the association between factors at the baseline (exposure) and Malaria positivity (outcome).

All statistical tests were two-tailed and statistical significance was assumed for a *p*-value < 0.05. Analyses were performed by using the SPSS 21.0 for Windows (SPSS Inc., Chicago, IL, USA).

### 2.2. Ethics Approval

Ethical approval of the protocol was achieved by District Health Authority in Beira, the Health District Direction (protocol reference: 189/17), Mozambique and (as this study used secondary data) informed patient consent was not required.

## 3. Results

A total of 701 adult HIV positive patients (*n* = 430, 61.3% females; *n* = 421, 60.0% under 40 years old) were enrolled in the study. The demographic and HIV related characteristics, overall and according to the malaria test result (positive versus negative), are summarized in Table 1. A positive malaria test was found in 232 (33.0%) patients. These patients, compared to the negative ones, were more frequently unemployed (*n* = 177; 76.3%), aged under 40 (*n* = 167; 72.0%), with a HIV positive partner (*n* = 52; 22.4%) and with a CD4 cell count <200 (*n* = 139; 59.9%), being all of these differences statistically significant (*p*-value: <0.05). By contrast, malaria negative patients resulted were more frequently receiving cotrimoxazole prophylaxis (*n* = 302; 64.3%), under ART (*n* = 360; 77.2%), and to be more regular in taking it ART (*n* = 261; 72.5%) (*p*-value: <0.05). No statistical differences between the two groups were observed for gender (*p*-value: 0.38) and HIV stage defined by the WHO (*p*-value: 0.38).

Table 2 reports the distribution of co-morbidities, stratified by malaria test result. Considering the whole sample, hypertension was found in 83 patients (11.8%), diabetes in 15 (2.1%), tuberculosis in 97 (13.8%) and other STIs in 188 (26.8%). Comparing the two groups, patients were more frequently affected by tuberculosis in the malaria positive group (n.61; 26.3%) (*p*-value: <0.05). Conversely, no statistically significant difference was highlighted between the two groups for hypertension, diabetes and other STIs.

The multivariate model considered the effects on malaria (dependent variable) of age (<40 years old), occupational status (unemployed), partner HIV positivity, being under ART, regularity of ART, CD4+ T cell count < 200, being under cotrimoxazole prophylaxis, irregularity of cotrimoxazole prophylaxis and presence of tuberculosis comorbidity. The following variables resulted predictive of malaria positivity (Table 3): age under 40 (O.R. = 1.56; 95%CI: 1.22–2.08), being unemployed (O.R. = 1.74; 95%CI: 1.24–2.21), irregularity of cotrimoxazole prophylaxis (O.R. = 1.42; 95%CI: 1.10–1.78), CD4 cell count < 200 (O.R. = 2.01; 95%CI: 1.42–2.32) and tuberculosis comorbidity (O.R. = 1.58; 95%CI: 1.17–2.79).

## 4. Discussion

This cross-sectional study aimed to estimate the prevalence of malaria and to identify its predictors in HIV patients accessing to the health center of São Lucas of Beira, an endemic malaria area. Malaria and HIV are two of the most challenging global health issues for developing countries, especially for Mozambique, where malaria accounts for 29% of all deaths, closely followed by AIDS, responsible of 27% of the mortality in the general population [13,14]. Moreover, it is well know that these two infectious diseases are closely linked each other. In HIV patients, during malaria co-infection, it has been reported to cause an increase in plasma HIV-1 RNA levels and a decline in the CD4+ T cell count [15]. On the other hand, HIV co-infection is associated with increased mortality in areas of stable malaria transmission, making both malaria severity and HIV important risk factors for death [16].

However, to the best of our knowledge, only a few studies have evaluated the epidemiological aspects of HIV and Malaria co-infection in Mozambique [12,13,14,15,16,17], and none of them have explored the HIV non-hospitalized population.

Malaria prevalence in our study population was 33.0%, being higher when compared to the 25.9% reported among HIV patients hospitalized at Central Hospital of Beira and to the 9.8% of HIV patients hospitalized at Central Hospital of Maputo in 2008 [17]. In the same direction, our study documented a higher malaria occurrence, if compared to similar studies conducted in other developing countries, such as Ghana (11.75%) [18], and Nigeria (18.5%) [19]. These differences could be due to the different study periods, considering that malaria transmission is seasonal, and has different endemicity levels.

Moreover, although it is well known than HIV patients are more susceptible to malaria, in this study we identified a further more vulnerable group, consisting of young and unemployed patients with a low CD4 cell count, irregular in taking cotrimoxazole prophylaxis and with a previous history of tuberculosis. This evidence is in line with the current literature [20,21]. In fact, correlation between age and level of malaria transmission is well known also among HIV negative patients: IgG levels, tending to increase with the age [22,23,24], influence the severity of malaria and immune response.

Again, according to the literature, the patients enrolled in our study undergoing regularly to ART were less susceptible to malaria. This effect could be explained by the non-adherence to ART that can increase the risk of opportunistic infection such as malaria, being included in the list of AIDS-related opportunistic infections by CDC since 2009 [25,26]. Likewise, also cotrimoxazole prophylaxis was just described as an important factor in reducing malaria incidence [12] and our data confirmed this hypothesis. Of interest, WHO recommends to stop cotrimoxazole in clinically stable patients with evidence of immune recovery and/or viral suppression under ART, while it should be continued in patients living in areas with high malaria and bacterial infection prevalence [27].

Another aspect emerging from our data is the role played by tuberculosis and health determinants such as socio-economic status in the interaction between HIV and malaria [28]. In fact, it is clear that poverty represents one of the major obstacle to the global burden control, leading to unfavourable outcomes most of all in these categories of patients [29,30,31]. Finally, our study also confirmed that having a HIV positive partner is a risk factor for malaria, since the behavioural determinant could be correlated both in the HIV transmission between the couple, the adherence to the ART therapy, the prophylaxis with cotrimoxazole and the proper use of the mosquito net [32,33,34,35,36,37]. On this basis, an appropriate couple-based care strategy should be integrated and implemented at the same time HIV behavioural and medical interventions. However, a further comparison with HIV negative patients could have been useful in order to better understand of the role of the HIV infection itself in the differences reported. Unfortunately, information on HIV negative patients were not available from our database.

The major limitations of this study are related to the cross-sectional design and to the typology of patients included in the study: since the study enrolled non-hospitalized patients, the possibility of self-medication for malaria could have biased the prevalence detected. In addition, the lack of data on pregnancy status, haemoglobin values and other health outcomes, did not allow a more comprehensive interpretation of results, so when HIV and malaria infection occur together a higher risk of complications should be considered.

Our findings allowed us to identify the profile of HIV patient needing more medical attention: young adults, unemployed, with a low CD4 cell count and irregularly accessing to ART and cotrimoxazole prophylaxis.

## 5. Conclusions

Despite the noted limitations, our study documented a very high malaria prevalence in a population of HIV subjects accessing an out-patients center in Mozambique, allowing us, at the same time, to better understand the profile of these patients that are highly vulnerable to co-infection. These findings could assist decision makers in efforts to plan HIV and malaria prevention interventions in low income countries and in settings with high endemicity. In particular, a more extensive use of cotrimoxazole for preventing and protecting opportunistic infections associated to malaria should be advocated with regard to young and unemployed HIV patients.

## Figures and Tables

**Table 1:** Demographic and HIV related characteristics of 701 HIV positive patients, stratified by malaria test results.

	Total	Malaria Test Result		*p*-Value
		Positive	Negative	
	*n*. 701 (100%)	*n*. 232 (33.0%)	*n*. 469 (77.0%)	
Sex				
Female	430 (61.3)	137 (59.0)	293 (62.5)	0.38
Male	271 (38.7)	95 (41.0)	176 (37.5)	
Age				
<40 years	421 (60.05)	167 (72.0)	254 (54.1)	<0.05
≥ 40 years	280 (39.95)	65 (28.0)	215 (45.9)	
Occupation				
Unemployed	350 (49.92)	177 (76.3)	173 (36.8)	<0.05
Employed	351 (50.07)	55 (23.7)	296 (63.2)	
Partner’ HIV status				
Positive	79 (11.3)	52 (22.4)	27 (5.8)	<0.05
Negative	281 (40.0)	110 (47.4)	171 (36.4)	
Missing	341 (48.7)	70 (30.2)	271 (57.8)	
HIV Stage by WHO				
I-II	322 (45.93)	112 (48.2)	210 (44.7)	<0.05
III-IV	379 (54.07)	120 (51.8)	259 (55.3)	
ART				
Yes	463(66.1)	103 (44.3)	360 (77.2)	<0.05
No	238 (33.9)	129 (55.7)	109 (22.8)	
Regularity of ART				
Yes	271 (58.53)	10 (19.4)	261 (72.5)	<0.05
No	192 (41.47)	93 (80.6)	99 (27.5)	
CD4+ T cell count				
< 200	245 (34.9)	139 (59.9)	106 (22.6)	<0.05
>200	456 (65.1)	93 (40.1)	363 (77.4)	
Cotrimoxazole prophylaxis				
Yes	353 (50.3)	51 (22.0)	302 (64.3)	<0.05
No	348 (49.7)	181 (88.0)	167 (35.7)	
Regularity of cotrimoxazole prophylaxis				
Yes	251 (71.10)	10 (19.6)	241 (79.8)	<0.05
No	102 (28.90)	41(80.4)	61 (20.2)	

**Table 2:** Distribution of co-morbidities among the 701 HIV positive patients enrolled, stratified by Malaria test results.

	Total	Malaria Test Result		*p*-value
		Positive	Negative	
	*n* = 701 (100%)	*n* = 232 (33.0%)	*n* = 469 (77.0%)	
Hypertension				
Yes	83 (11.84)	21 (9.0)	62 (13.2)	0.10
No	618 (88.15)	211 (91.0)	407 (86.8)	
Diabetes				
Yes	15 (2.13)	3 (1.3)	12 (2.3)	0.41
No	686 (97.86)	229 (98.7)	457 (97.7)	
Tuberculosis				
Yes	97 (13.83)	61 (26.3)	36 (7.7)	<0.05
No	604 (86.16)	171 (73.7)	433 (92.3)	
Other STI *				
Yes	188 (26.81)	71 (30.6)	117 (24.9)	0.13
No	513 (73.18)	161 (69.4)	352 (75.1)	

* STI: sexual transmitted infections.

**Table 3:** Predictors of positivity at Malaria Test in HIV positive patients.

Characteristics	Univariate Analysis OR	Multivariate Analysis Adj-OR
Age < 40	1.28 (1.06–1.78)	1.56 (1.22–2.08) *
Partners HIV positivity	0.36 (0.08–0.83)	0.42 (0.08–1.03)
Unemployed	1.85 (1.35–2.45)	1.74 (1.24–2.21) *
ART	0.64 (0.38–0.78)	0.74 (0.50–1.03)
Regularity of ART	0.75 (0.49–0.92)	0.51 (0.28–0.85)
CD4+ T cell count < 200	1.91 (1.34–2.19)	2.01 (1.42–2.32) *
Cotrimoxazole prophylaxis	0.53 (0.41–0.83)	0.58 (0.43–0.90)
Irregularity of Cotrimoxazole prophylaxis	1.80 (1.50–2.00)	1.42 (1.10–1.78) *
Tuberculosis	1.31 (1.06–1.66)	1.58 (1.17–2.79) *

* Statistically significant values; OR: Odds Ratio; Adj-OR: Adjusted Odds Ratio; ART: antiretroviral therapy.
